# Laparoscopic versus open right hemicolectomy in colon carcinoma: A propensity score analysis of the DGAV StuDoQ|ColonCancer registry

**DOI:** 10.1371/journal.pone.0218829

**Published:** 2019-06-27

**Authors:** Christian Jurowich, Sven Lichthardt, Caroline Kastner, Imme Haubitz, Andre Prock, Jörg Filser, Christoph-Thomas Germer, Armin Wiegering

**Affiliations:** 1 Department of General, Visceral and Thoracic Surgery Kreiskliniken Altötting / Burghausen, Altötting, Germany; 2 Department of General, Visceral, Vascular and Pediatric Surgery, University Hospital, University of Wuerzburg, Wuerzburg, Germany; 3 Comprehensive Cancer Centre Mainfranken, University of Wuerzburg Medical Center, Wuerzburg, Germany; 4 Department of Biochemistry and Molecular Biology, University of Wuerzburg, Wuerzburg, Germany; UKSH Campus Lübeck, GERMANY

## Abstract

**Objective:**

To assess whether laparoscopy has any advantages over open resection for right-sided colon cancer.

**Summary background data:**

Right hemicolectomy can be performed using either a conventional open or a minimally invasive laparoscopic technique. It is not clear whether these different access routes differ with regard to short-term postoperative outcomes.

**Methods:**

Patients documented in the German Society for General and Visceral Surgery StuDoQ|ColonCancer registry who underwent right hemicolectomy were analyzed regarding early postoperative complications according to Clavien-Dindo (primary endpoint), operation (OP) time, length of postoperative hospital stay (LOS), MTL30 and number of lymph nodes retrieved (secondary endpoints).

**Results:**

A total of 4.997 patients were identified as undergoing oncological right hemicolectomy without additional interventions. Of these, 4.062 (81.3%) underwent open, 935 (18.7%) laparoscopic surgery. Propensity score analysis showed a significantly shorter LOS (OR: 0.55 CI 95%0.47-.64) and a significantly longer OP time (OR2.32 CI 1.98–2.71) for the laparoscopic route. Risk factors for postoperative complications, anastomotic insufficiency, ileus, reoperation and positive MTL30 were higher ASA status, higher age and increasing BMI. The surgical access route (open / lap) had no influence on these factors, but the laparoscopic group did have markedly fewer lymph nodes retrieved.

**Conclusion:**

The present registry-based analysis could detect no relevant advantages for the minimally invasive laparoscopic access route. Further oncological analyses are needed to clarify the extent to which the smaller lymph node harvest in the laparoscopic group is accompanied by a poorer oncological outcome.

## Introduction

Colorectal cancer is worldwide the most common malignant disease of the gastrointestinal tract and the second to third most common tumor disease with over one million new diagnoses and 500.000 deaths annually [1]. Approximately 40% of all colorectal cancers are located in the right hemicolon. In recent years, „complete mesocolic excision”(CME), first described by Hohenberger et al in 2009, has dramatically changed the surgical procedure for colorectal cancer [2]. At the same time, the laparoscopic technique for colorectal cancer has shown a marked growth in popularity [3]. Laparoscopic resection of the left colon and rectum is now standardized and achieves the same oncological results as open resection with lower perioperative morbidity [4].

Far fewer data are available on oncological right hemicolectomy. Large randomized studies have focused on left-sided resection, while numerous technical variations are available for minimally invasive right hemicolectomy: CME, ligation of the vessels, and various methods for creating anastomoses [5–8]. Moreover, the conversion rate for oncological right hemicolectomy (up to 18.9%) is relatively high compared to that of left-sided resection [9]. A recent meta-analysis encompassing 3307 patients from two randomized controlled trials (RCT) (n = 211 patients) and 24 non-RCT (n = 3096 patients) showed that the primary endpoints 30-day mortality (RR 0.45; 95% CI 0.21–0.93, p = 0.031) and overall complication rate (RR 0.81; 95% CI 0.70–0.95, p = 0.007) were lower for laparoscopic patients [10]. Secondary endpoints such as anastomotic insufficiency, blood loss, length of postoperative hospital stay (LOS), and 5-year survival favored laparoscopy or did not differ significantly. The number of lymph nodes retrieved—at about 16 in each group—did not differ significantly either, though the number was markedly lower than in the literature on open right CME [2, 11–14]. Valid data are not available on long-term survival after oncological laparoscopic right hemicolectomy. Almost all patients included in the meta-analysis were operated on before introduction of CME in 2009. A more recent meta-analysis specifically investigating CME depending on surgical procedure (1377 lap vs. 1265 open) also found no advantages or disadvantages for laparoscopy except for a lower wound infection rate with longer operation (OP) time [15]. Thus the demonstrated advantage of left-sided colon and rectum resections continues to be of doubtful applicability to right-sided resections. To investigate this using „real world”data from the developed world, we performed a propensity score analysis of patient data in the DGAV StuDoQ|ColonCancer registry.

## Materials and methods

The StuDoQ|ColonCancer registry is a prospectively documented database for colon cancer surgery established by the DGAV in January 2010 (www.dgav.de/studoq, www.en.studoq.de). It was designed to facilitate assessment of the quality of and risk factors associated with colon cancer surgery in Germany. Its informed consent and data safety procedures were approved by the Society for Technology, Methods, and Infrastructure for Networked Medical Research (http://www.tmf-ev.de), its publication guidelines were established by the DGAV (http://www.dgav.de/studoq/datenschutzkonzept-und-publikationsrichtlinien.html). Data from participating centers are prospectively entered in pseudonymized form using a browser-based tool and subjected to automatic plausibility controls. Validation by cross-checking with institutional medical controlling data is part of the annual certification process. For the present study, all cases of right or extended right hemicolectomy were identified from the StuDoQ|ColonCancer registry and relevant demographic data, comorbidities, and information on operations, histology, and perioperative course were extracted in anonymized form for analysis. Basic registry structures are comparibel to the StuDoQ|Pancreas registry [16].

Anastomotic leakage [17, 18], surgical site infection [19], Clavien-Dindo classification (CDC) [5], burst abdomen, reoperation, and in-hospital mortality were defined as either present or absent. Additional postoperative parameters assessed were need for unplanned postoperative ventilation lasting more than 48 hours, pneumonia, LOS, and readmission. Overall postoperative morbidity was summarized according to the CDC as none (CDC 0), minor (CDC 1–2), major (CDC 3a-4), and death (CDC 5).

Statistical analysis was performed with a two-sided significance level of 0.05. Scale variables were expressed as median and range and categorical parameters as absolute frequency and percentage. Univariate analysis was performed using the Chi-square test for categorial variables and the Mann-Whitney test for rational variables.

Multivariable analysis was by Cox regression. All variables with a p-value <0.1 in univariate analysis were included in the multivariate analysis. Propensity scoring was used to control for the influence of observed data on choosing the OP method. For stratification five parameters were chosen to control for according to logistic regression model as described elsewhere: loss of weight, disseminated tumor disease, liver metastasis, diabetes, and clinical tumor stage (grouped T0-T2 vs. T3-T4). 32 possible combination groups were possible in the propensity score calculation. Of this 26 gave reliable results. Strata with less than 4 members in each group (open / lap) were omitted (138 of 4883 patients). The logistic model for matched data was used [20].

Hospitals providing patient data are listed in S1 Table.

## Results

Of 4.997 patients included in this study, 4.062 (81.3%) underwent an open, 935 (18.7%) a laparoscopic procedure (with a 16.5% conversion rate, see below) (S1 Fig). 46.95% of the patients were male, mean age at the time of surgery was 72.9 years (range 22–98 years). Table 1 summarizes patient characteristics by surgical access route. Patients who underwent laparoscopic surgery were significantly younger (73.2 years vs. 71.7 years p<0.001), had lower ASA scores, better ECOG scores, and had fewer and less severe prior illnesses; patient BMIs did not differ significantly (Table 1 and S2A Fig). The proportion of patients who underwent laparoscopic surgery increased over time. The conversion rate of laparoscopically begun procedures was 16.5%, a rate that remained stable over time (S2B Fig). In the laparoscopic group, tumors were significantly more often located in the cranial portion of the right hemicolon, they had a clinically lower T stage, and were significantly less likely to have metastases (Table 1).

**Table 1:** Preoperative patient characteristics by type of surgical approach.

	Variable	Open (n = 4.062)	Laparoscopic (n = 935)	p-value
	Age, average +/- SD. yrs	73.2 +/- 10.8	71.7+/- 10.7	<0.0001
Sex	male, n (%)	1915 (81.63)	431 (18.37)	n.s.
	female, n (%)	2147 (80.99)	698 (19.01)	
	BMI (kg/m^2^)	26.77 (+/-5.18)	26.72 +/- 4.66	n.s.
	Smoking, n (%)	275 (7.54)	57 (6.86)	n.s.
ASA status, n (%)	1	182 (4.5)	68 (7.3)	<0.0001
	2	1721 (42.4)	486 (52)	
	3	2004 (49.3)	364 (38.9)	
	4	150 (3.7)	17 (1.8)	
	5	5 (0.12)	0 (0)	
Functional status, n (%)	Independent	3554 (87.5)	852 (91.1)	0.006
	Partially dependent	437 (10.8)	71 (7.6)	
	Totally dependent	71 (1.7)	12 (1.3)	
Comorbidities; n (%)	Diabetes (IDDM and NIDDM)	986 (23.8)	172 (18.4)	0.0012
	Heart failure (NYHA I-IV)	902 (22.9)	184 (20.2)	n.s.
	History of severe COPD	280 (6.9)	58 (6.2)	n.s.
	Chronic steroid use	62 (1.5)	7 (0.8)	0.048
	Dialysis	37 (0.9)	5 (0.5)	n.s.
	Disseminated cancer	248 (6.1)	42 (4.5)	0.05
	Weight loss (>10% body weight)	63 (7.9)	20 (14.5)	0.018
	Alcohol abuse	19 (2.4)	7 (5.1)	n.s.
UICC stage	1	875 (21.7)	297 (31.9)	<0.0001
	2	1546 (38.3)	305 (32.8)	
	3	1132 (28)	253 (27.2)	
	4	484 (12)	75 (8.1)	
pT-stage	T0-2	1.006 (24.8)	342 (36.7)	<0.0001
	T3/4	3.046 (75.2)	589 (63.3)	
pN-stage	N0	2508 (61.9)	617 (66)	0.019
	N1/2	1544 (38.1)	318 (34)	
R-stage	R0	3939 (98.06)	927 (99.25)	0.016
	R1/2	78 (1.04)	7 (0.75)	

Table 2 shows the surgical characteristics of the study population. The OP time was significantly shorter in the open group (129.7 min vs 148.4 min; p<0.0001). The open group was significantly more likely to undergo extended right hemicolectomy. The percentage of stapled anastomoses and the number of intraoperative transfused erythrocyte concentrates did not differ. The open group underwent CME significantly less often, while the average number of lymph nodes retrieved per patient did not differ (open: 25.1 lymph nodes [LN] vs lap: 24.7 LN; p0.41). There was however a trend for laparoscopic patients to have fewer than 12 lymph nodes retrieved, though the difference was not significant (2.4% vs 1.55%; p = 0.17).

**Table 2:** Unadjusted postoperative variables by type of surgical approach.

	Variable	Open (n = 4.062)	Laparoscopic (n = 935)	p-value
	Total operation time (min)	129.65 +/- 48.6	148.4 +/- 51.7	<0.0001
	Extended resection; n (%)	588 (13.8)	71 (8.7)	<0.0001
Anastomosis	Hand-sewn	2886 (67.7))	544 (67)	0.8
	stapler	1378 (32.3)	268 (33)	
CME; n (%)	YES	3150 (82.8)	697 (90.7)	<0.0001
	NO	656 (17.2)	71 (9.2)	
	Anastomotic leak, n (%)	146 (3.6)	34 (3.6)	0.97
	Postoperative ileus, n (%)	159 (3.9)	42 (4.5)	0.42
	Return to the operating room, n (%)	303 (9.9)	87 (9.3)	0.5
	Superficial site infection, n (%)	520 (12.8)	103 (11)	0.13
	Internal complication	556 (13.7)	88 (9.41)	0.0003
	Postoperative hemorrhage, n (%)	70 (1.7)	15 (1.6)	0.8
	Transfusion, n (%)	91 (2.24)	13 (1.39)	0.085
Clavien-Dindo, n (%)	0	2579 (63.71)	658 (70.37)	0.014
	1	195 (4.82)	40 (4.28)	
	2	561 (13.86)	95 (10.16)	
	3a	194 (4.79)	40 (4.28)	
	3b	290 (7.16)	62 (6.63)	
	4a	90 (2.22)	15 (1.6)	
	4b	35 (0.86)	6 (0.64)	
	5	104 (2.57)	19 (2.03)	
	Postoperative length of stay (days)	12.5+/-9.3	11.4 +/-8.8	<0.0001
	MTL 30	385 (9.5)	72 (7.7)	0.83
	Mortality	128 (3.15)	24 (2.57)	0.34
	Number of lymph nodes retrieved	25.1 +/-12.15	24.97 +/- 11.81	0.41

Univariate analysis of postoperative course revealed the laparoscopic group to have a significantly shorter LOS, fewer internal complications, a lower MTL30, and fewer major CDC complications. No differences were found between the two procedures regarding surgery-related complications such as postoperative hemorrhage, ileus, anastomotic insufficiency or wound healing disturbances. (Table 2)

In multivariate analysis the surgical access route was not found to be a predictive factor for complications, anastomosis leakage, re-operation, postoperative ileus or positive MTL30. Laparoscopy was positive correlated with significant reduced length of stay and prolonged operation time (S2 Table).

Because the basic variables differed significantly between laparoscopic and open surgery patients, we performed a propensity score matched analysis. This again showed the laparoscopic access route to have a significantly shorter LOS (OR: 0.55 CI 95%0.47-.64) and significantly longer OP time (OR2.32 CI 1.98–2.71). Risk factors for postoperative complications such as anastomotic insufficiency, ileus, reoperation and MTL30 were ASA, age and BMI. The surgical access route (lap / open) had no influence on these factors.

**Fig 1:**
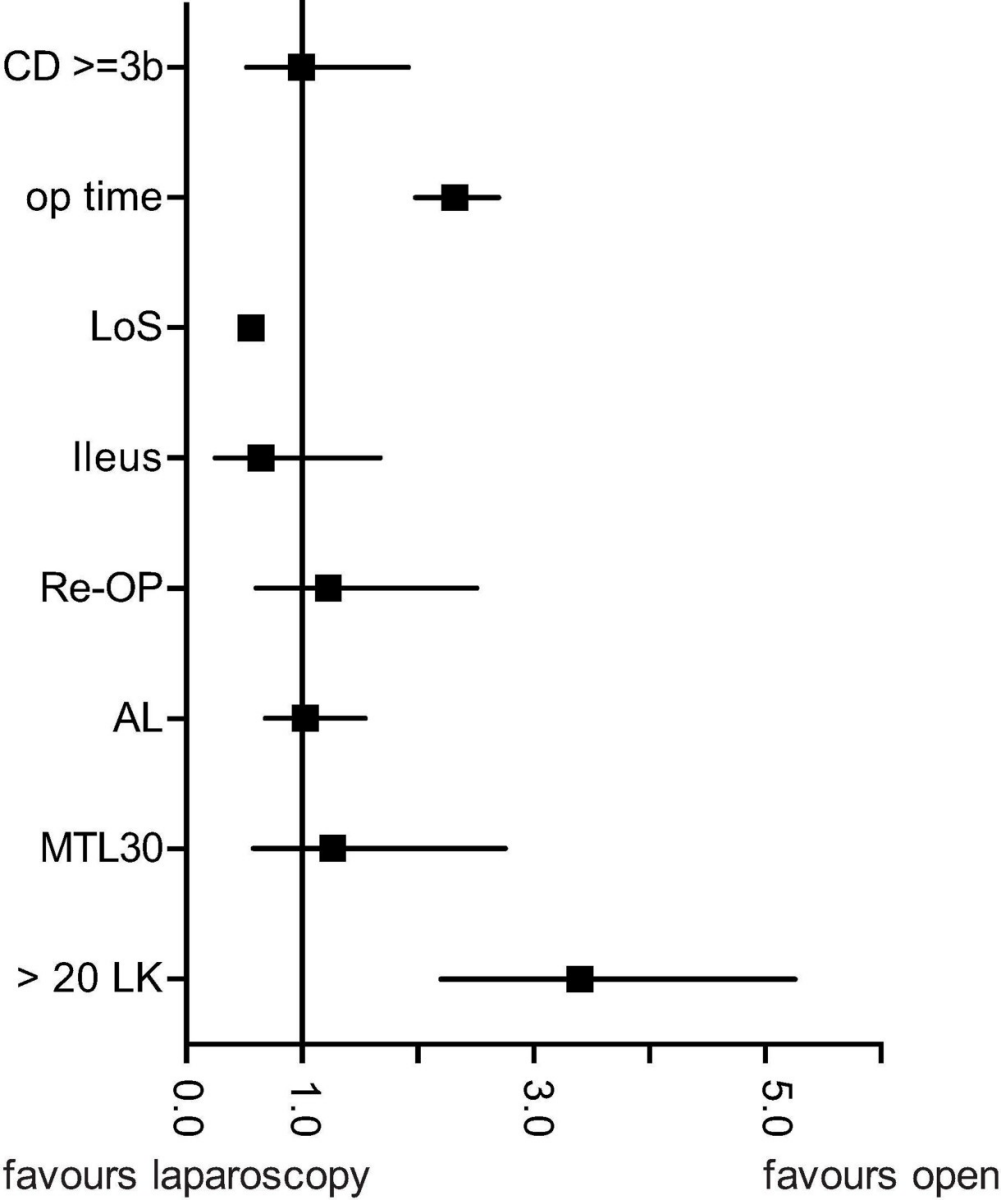
Adjusted odds ratios for outcomes by type of surgical approach.

The number of lymph nodes retrieved served as surrogate parameter for oncological quality of the resected tissue. Because very few patients (n = 112) had less than 12 lymph nodes retrieved, 20 lymph nodes retrieved was used as cut-off. The literature shows that patients with >20 lymph nodes retrieved have significantly better oncological outcomes than those with <20 lymph nodes. In the present study, patients in the open group were significantly more likely to have ≥20 lymph nodes retrieved (OR: 3.45 CI95%: 2.22–5.26; p<0.0001) (Tables 3–5 and S2 and Fig 1).

**Table 3:** Propensity score best-fit model for complication, operation time and length of stay (*** = p<0.0001).

	Clavien-Dindo > = 3b		Operation time		Length of stay	
	OR; (95% CI)	p-value	OR; (95% CI)	p-value	OR (95% CI)	p-value
Open Laparoscopic	Ref 0.99 (0.51–1.92)	0.99	Ref 2,32 (1.98–2.71)	0.000***	Ref 0.56 (0.47–0.65)	0.000***
Hemicolectomy Ext. Hemicolectomy	Ref 1.09 (0.84–1.41)	0.52	Ref 1,46 (1,22–1,75)	0.000***	Ref 1.16 (1.45–1.79)	0.11
ASA I (per 1 ASA category)	Ref 1.8 (1.55–2.1)	0.000***	Ref 1.11 (1.01–1.23)	0.035	Ref 1.62 (1.45–1.79)	0.000***
BMI per 5kg/m^2^	Ref 1.13 (1.04–1.22)	0.004	Ref 1.27 (1.19–1.35)	0.000***	Ref 1.09 (1.03–1.16)	0.004
Age per 10 years	Ref 1.13 (1.03–1.25)	0.011	Ref 0.9 (0.85–0.95)	0.001	Ref 1.34 (1.26–1.43)	0.000***

**Table 4:** Propensity score best-fit model for anastomotic leakage, postoperative ileus and re-operation rate. (*** = p<0.0001).

	Anastomotic leak		Postoperative ileus		Re-operation	
	OR; (95% CI)	p-value	OR; (95% CI)	p-value	OR (95% CI)	p-value
Open Laparoscopic	Ref 1.03 (0.69–1.55)	0.89	Ref 0.65 (0.25–1.67)	0.37	Ref 1.23 (0.6–2.51)	0.57
Hemicolectomy Ext. Hemicolectomy	Ref 1.21 (0.78–1.87)	0.4	Ref 1.11 (0.73–1.69)	0.62	Ref 1.18 (0.89–1.56)	0.25
ASA I (per 1 ASA category)	Ref 1.75 (1.37–2.23)	0.000***	Ref 1.22 (0.97–1.53)	0.086	Ref 1.57 (1.34–1.83)	0.000***
BMI per 5kg/m^2^	Ref 1.14 (0.99–1.31)	0.058	Ref 1.13 (0.99–1.29)	0.069	Ref 1.19 (1.09–1.3)	0.000***
Age per 10 years	Ref 0.88 (0.74–1.03)	0.11	Ref 1.03 (0.89–1.21)	0.63	Ref 099 (0.9–1.1)	0.97

**Table 5:** Propensity score best-fit model for MTL30 and fewer than 20 lymph nodes retrieved (*** = p<0.0001).

	MTL30 positive		> 20 LK	
	OR; (95% CI)	p-value	OR; (95% CI)	p-value
Open Laparoscopic	Ref 1.26 (0.58–2.76)	0.56	Ref 0.29 (0.19–0.45)	0.000***
Hemicolectomy Ext. Hemicolectomy	Ref 1.00 (0.73–1.37)	0.98	Ref 1.35 (1.12–1.62)	0.001
ASA I (per 1 ASA category)	Ref 2.43 (1.13–2.92)	0.000***	Ref 0.79 (0.72–0.88)	0.000***
BMI per 5kg/m^2^	Ref 1.08 (0.98–1.19)	0.11	Ref 0.98 (0.93–1.04)	0.55
Age per 10 years	Ref 1.27 (1.13–1.43)	0.000***	Ref 0.82 (0.77–0.87)	0.000***

## Discussion

The present study is to our knowledge the second registry-based investigation worldwide to compare open and minimally-invasive oncological right hemicolectomy. It is first such study in Germany.

It is noteworthy that under real-world conditions in the StuDoQ|ColonCancer registry the proportion of patients with right-sided colon carcinoma who underwent minimally invasive surgery (18.7%) is smaller than in similar groups in other registries. The study of Bosker et al, for example, reports a proportion of 44.3% [21]. Moreover, the 63.3% of T3/4 tumors is clearly lower than the 73.5% in the Dutch analysis.

This smaller rate of minimally invasive surgery in the StuDoQ|ColonCancer registry is due presumably to the fact that since 2009 CME has become increasingly standardized in Germany while also becoming increasingly the go-to procedure for these tumors [2]. While a recent review / meta-analysis by Negoi et al concludes that the minimally invasive access route delivers the same surgical quality with regard to the CME as the conventional open approach, in Germany there is no agreement on this point [22]. An ongoing study (still in the recruiting phase) on standardization of laparoscopic CME (DRKS-ID: DRKS00012369) is once again examining this very point.

Although the present study cannot analyze the quality of CME based on registry data, the lymph nodes harvests of laparoscopic versus open surgery (>20 LN OR 3.4 CI: 2.2–5.3; p<0.001) indicates less extended lymph and soft tissue dissection in the laparoscopic group. This stands in contrast to the data of Negoi [22] et al, which found no difference in the size of the lymph nodes harvest in an analysis of one RCT and 9 non-RCT, although in the former an average of only 22 lymph nodes were retrieved with minimally invasive colectomy versus 21 with open colectomy [23]. Their study however used the Japanese D3 lymph adenectomy as standard. A study comparing CME with central ligation of the vessels and systematic lymph adenectomy to the Japanese D3 lymphadenectomy, however, clearly showed the latter to be inferior to CME [12]. The study of Negoi, therefore, does not represent the current surgical gold standard in the Western world.

In addition, the technique for laparoscopic right hemicolectomy has not been definitively standardized with regard to the creation of anastomoses. Although the rate of intracorporal anastomosis appears to be rising, as a rule the incision to retrieve the surgical specimen is made in the right abdomen and the anastomosis also constructed [24]. This incision is often only slightly smaller than a primary completely open access via a transverse right-sided upper abdomen laparotomy and therefore represents in the view of some surgeons a fundamental argument against the minimally invasive access route. This issue was examined in a recent retrospective multicenter propensity score analysis [24] that showed the intracorporal anastomosis technique–with indeed significantly longer OP times (p<0.0001)–had clear advantages for minimally invasive right hemicolectomy with regard to various clinical parameters (lower conversion rate [p = 0.01], shorter LOS [p = 0.02], and lower complication rate from discharge to 30 days post-OP [p = 0.04]).

In addition to the question of oncological quality, however, the perioperative results of the laparoscopic versus the open surgical procedure appear to be important. Although several studies have already demonstrated advantages for the laparoscopic access route, this often applies chiefly to left-sided colon resection.

The present study in fact confirms that patients who underwent minimally invasive surgery had a shorter LOS, fewer overall complications, a lower MTL 30, and a lower rate of severe CDC complications. If the two groups are compared with regard to patient characteristics, though, a clear selection bias must be assumed since the risk profile for the minimally invasive surgery patients is lower and the tumors locally less advanced. Thus severe cases continue to be more likely to undergo open surgery. The propensity score analysis therefore showed no advantages and—unlike the analysis of Bosker et al—no relevant differences between the two groups. Only the difference with regard to LOS remained unchanged. This difference can only be definitively resolved by a blind study. Here it must be said that the LOS we report (12.4 days versus 11.4 days) are very high compared to LOS reported elsewhere in the world. The cause for this may well lie in the German Diagnosis Related Groups (DRG) system and the continuing variations in perioperative management despite the acceptance of the ERAS concept.

Of note in our registry is the lack of a difference between the laparoscopic and open groups in 30-day mortality. Whereas the systematic meta-analysis of 3049 patients by Arezzo et al in 2015 could show a significant difference (1.2% lap vs. 3.4% open), our own analysis did not find a significant difference (2.57% lap vs. 3.15% open), this despite the possible selection bias [10]. Our own data also stand in contradiction to those of the Dutch study of Bosker et al. [21].

## Conclusion

The present registry-based analysis comparing laparoscopic and open right hemicolectomy in patients with colon carcinoma is by far the to-date largest study on this question. No relevant advantages could be found for the minimally invasive access route. The shorter LOS in the minimally invasive group should be interpreted with caution in view of the comparatively long LOS of both groups. Of crucial importance however is the significantly small harvest of lymph nodes retrieved from the laparoscopic group as surrogate marker for the oncological quality of the surgery. Further oncological analyses are needed to clarify the extent to which this smaller lymph nodes harvest is also accompanied by a poorer oncological outcome. In light of the recommendations of the German S3 guidelines for performing CME, therefore, laparoscopic right hemicolectomy should be regarded critically until an improvement is found in the data from sources other than clinical trials.

## Supporting information

S1 Fig Patient selection flow-chart. (DOCX)

S2 Fig Percentage of patients undergoing laparoscopic procedure dependent on clinical factors; (A) ASA score; (B) T stage.
(DOCX)

S1 Table Hospitals contributing data in the StuDoQ registry. (XLSX)

S2 Table Multivariate adjusted odd ratios for postoperative outcome parameters by access route. (DOCX)
