# Supplementary material for: Laparoscopic versus open right hemicolectomy in colon carcinoma: A propensity score analysis of the DGAV StuDoQ|ColonCancer registry
Source: PLoS One. 2019 Jun 27;14(6):e0218829. doi: 10.1371/journal.pone.0218829 (PMC6597089; doi:10.1371/journal.pone.0218829)
Supplement: S1 Fig — (DOCX) [file pone.0218829.s001.docx]

Figure 1 Patient selection

| As of 17 August 2017  16.151 patients  with colon cancer  registered in StuDoQ | | | |  |  |  |
| --- | --- | --- | --- | --- | --- | --- |
|  | |  | |  | 2.404 Patients excluded  due to lack of consent or missing essential data |  |
|  |  |  | |  |  |  |
| 13.747 patients | | | |  |  |  |
|  | |  | |  | 8.452 patients excluded for cancer location other than right-sided or cancer therapy was emergency treatment or non-surgical |  |
|  | |  | |  |  |  |
| 5.295 patients | | | |  |  |  |
|  | |  | |  | 298 Patients excluded due to concurrent liver resection (165), procedure not clear (133) |  |
|  |  |  | |  |  |  |
| 4.997 patients | | | |  |  |  |
|  | |  | |  |  |  |
|  | | | |  |  |  |
| open  (81.3%; n=4.062) | |  | | laparoscopic (18.7%; n=935) | |  |
