# Supplementary material for: Laparoscopic versus open right hemicolectomy in colon carcinoma: A propensity score analysis of the DGAV StuDoQ|ColonCancer registry
Source: PLoS One. 2019 Jun 27;14(6):e0218829. doi: 10.1371/journal.pone.0218829 (PMC6597089; doi:10.1371/journal.pone.0218829)
Supplement: S2 Fig — Percentage of patients undergoing laparoscopic procedure dependent on clinical factors; (A) ASA score; (B) T stage. (DOCX) [file pone.0218829.s002.docx]

Supp. Figure 2: Percentage of patients undergoing laparoscopic procedure dependent on clinical factors; (A) ASA score; (B) T stage
