# Supplementary material for: Laparoscopic versus open right hemicolectomy in colon carcinoma: A propensity score analysis of the DGAV StuDoQ|ColonCancer registry
Source: PLoS One. 2019 Jun 27;14(6):e0218829. doi: 10.1371/journal.pone.0218829 (PMC6597089; doi:10.1371/journal.pone.0218829)
Supplement: S2 Table — (DOCX) [file pone.0218829.s004.docx]

|  | Clavien-Dindo >=3b | | Operation time | | Length of stay | | Anastomotic leak | | Postoperative ileus | | Re-operation | | MTL30 positive | | > 20 LK | |
| --- | --- | --- | --- | --- | --- | --- | --- | --- | --- | --- | --- | --- | --- | --- | --- | --- |
|  | OR;  (95% CI) | p-val. | OR;  (95% CI) | p-val. | OR;  (95% CI) | p-val. | OR;  (95% CI) | p-val. | OR;  (95% CI) | p-val. | OR;  (95% CI) | p-val. | OR;  (95% CI) | p-val. | OR;  (95% CI) | p-val. |
| Open  Laparoscopic | Ref  0.92  (0.73-1.16) | 0.49 | Ref  2.18  (1.87-2.54) | <0.001 | Ref  0.54  (0.46-0.64) | <0.001 | Ref  1.11  (0.76-1.65) | 0.58 | Ref  1.2  (0.85-1.71) | 0.3 | Ref  1.02  (0.8-1.31) | 0.85 | Ref  0.97  (0.74-1.26) | 0.34 | Ref  0.83  (0.72-0.96) | 0.014 |
| Hemicolectomy  Ext. Hemicole. | Ref  1.08  (0.84-1.39) | 0.53 | Ref  1.48  (1.25-1.76) | <0.001 | Ref  1.13  (0.95-1.35) | 0.16 | Ref  1.19  (.078-1.8) | 0.43 | Ref  1.07  (0.71-1.62) | 0.75 | Ref  1.2  (0.92-1.56) | 0.18 | Ref  1.01  (0.75-1.36) | 0.96 | Ref  1.39  (1.16-1.66) | <0.001 |
| ASA I  (per 1 ASA cat.) | Ref  1.86  (1.6-2.15) | <0.001 | Ref  1.09  (0.98-1.2) | 0.1 | Ref  1.62  (1.46-1.79) | <0.001 | Ref  1.7  (1.35-2.15) | <0.001 | Ref  1.25  (1.00-1.55) | 0.05 | Ref  1.6  (1.38-1.86) | <0.001 | Ref  2.58  (2.17-3.07) | <0.001 | Ref  0.8  (0.72-0.88) | <0.001 |
| BMI  per 5kg/m^2^ | Ref  1.13  (1.04-1.22) | 0.002 | Ref  1.27  (1.19-1.34) | <0.001 | Ref  1.09  (1.03-1.16) | 0.002 | Ref  1.1  (0.97-1.27) | 0.14 | Ref  1.11  (0.98-1.27) | 0.1 | Ref  1.2  (1.1-1.3) | <0.001 | Ref  1.09  (0.99-1.19) | 0.07 | Ref  0.99  (0.93-1.05) | 0.68 |
| Age  per 10 years | Ref  1.12  (1.02-1.23) | 0.016 | Ref  0.92  (0.87-0.97) | <0.001 | Ref  1.33  (1.25-1.41) | <0.001 | Ref  0.87  (0.75-1.01) | 0.059 | Ref  1.03  (0.89-1.19) | 0.71 | Ref  1.0  (0.9-1.1) | 0.93 | Ref  1.26  (1.13-1.41) | <0.001 | Ref  0.83  (0.78-0.88) | <0.001 |
